# Microsporidium Keratitis After Hurricane Francine: A Case Report

**DOI:** 10.1155/crop/5069667

**Published:** 2026-01-26

**Authors:** Zachary A. Dwyer, Stephen V. Lau

**Affiliations:** ^1^ Kirksville College of Osteopathic Medicine, A.T. Still University, Kirksville, Missouri, USA, atsu.edu; ^2^ Northshore Eye Associates, Hammond, Louisiana, USA

## Abstract

Microsporidial ocular infections are being increasingly reported, especially in temperate climates. In this case report, we describe five patients who presented to a private ophthalmology practice in Southeastern Louisiana in 2024, all within 3 months of Hurricane Francine landing. The patients’ ages ranged from 5 to 23 and included two female and three male patients. All patients except one had exposure to contaminated water, and microsporidia were detected via PCR in all patients. Microsporidial keratoconjunctivitis was diagnosed based on typical clinical features. The patients were all effectively treated with a course of moxifloxacin and subsequent fluorometholone. This paper reports the typical clinical course of microsporidial keratoconjunctivitis and offers an effective management approach to this condition.

## 1. Introduction

Microsporidia are obligate intracellular spore‐forming parasites, now known to be specialized fungi [1]. Once thought to only cause severe disease in immunocompromised patients, cases of immunocompetent patients with infections of the gastrointestinal tract, lungs, and eyes have been reported [2–4]. The fungus is typically contracted from exposure to contaminated soil and water [5].

Diagnosis has largely been based on detecting spores via light microscopy or electron microscopy [2]. However, newer polymerase chain reaction (PCR) testing has allowed for quicker detection of microsporidia with lower costs [6]. Ocular microsporidiosis may present as keratoconjunctivitis or stromal keratitis. Keratoconjunctivitis typically appears as raised punctate gray–white corneal lesions that evolve to nummular scars before resolution [7]. On the other hand, stromal keratitis results in diffuse stromal infiltrates and endothelial exudates [8, 9]. Various treatment regimens have been used including albendazole with fumagillin and voriconazole with gatifloxacin [10, 11].

This case report is aimed at presenting five cases of microsporidial keratitis that occurred in Southeastern Louisiana, potentially related to Hurricane Francine which landed in September 2024.

## 2. Case Presentation

The following five cases presented to a private practice located in Southeastern Louisiana. All five cases presented within 3 months of Hurricane Francine in 2024. The first case presented is a representative of the course of infection and exposure risk factors in four kids (ages 5, 10, 10, and 11). The 5‐year‐old female was lost to follow‐up after initial treatment, but the other three children completed the entire treatment regimen. The second case presents a 23‐year‐old female who may have had indirect exposure through her job as an aquatics instructor for children. The presentation of the other three cases has been tabulated and is listed after Case 2 (Table 1). None of the patients reported being immunocompromised. No written consent has been obtained from the patients, as there is no patient‐identifiable data included in this case report.

**Table 1:** Initial presentations of the three other cases.

**Initial presentations of other cases**			
**Age/sex**	**Visual acuity**	**Intraocular pressure (mmHg)**	**Exam findings**
5F	20/30 OS	18	Trace injection, scattered SEIs
11M	20/20 OS	20	1+ injection, 1+ chemosis, scattered SEIs
10M	20/30 OD	13	1+ injection, scattered SEIs

### 2.1. Case 1

**Figure 1:**
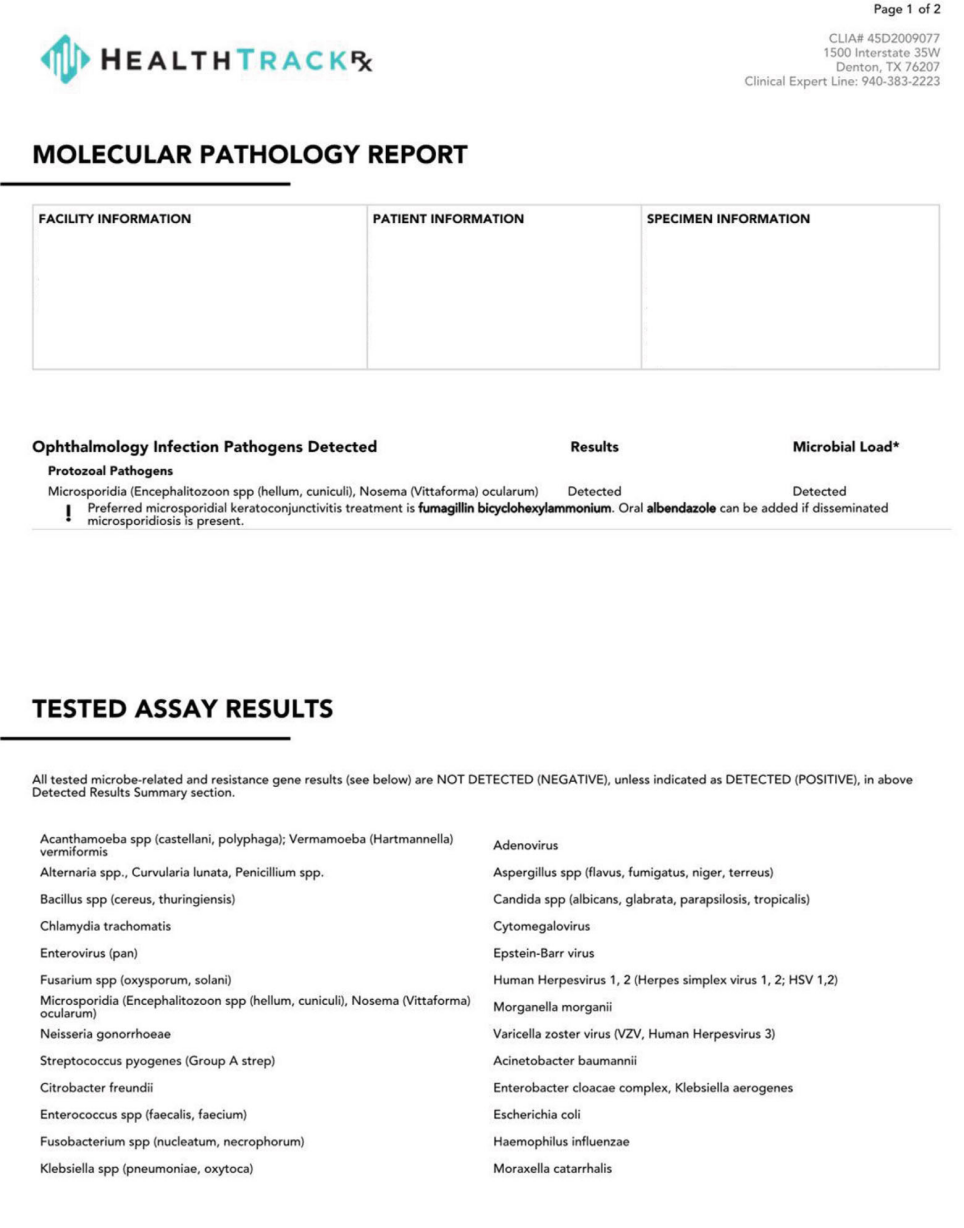
Representative image of PCR results indicating positive results for microsporidia and pertinent negatives such as adenovirus.

**Figure 2:**
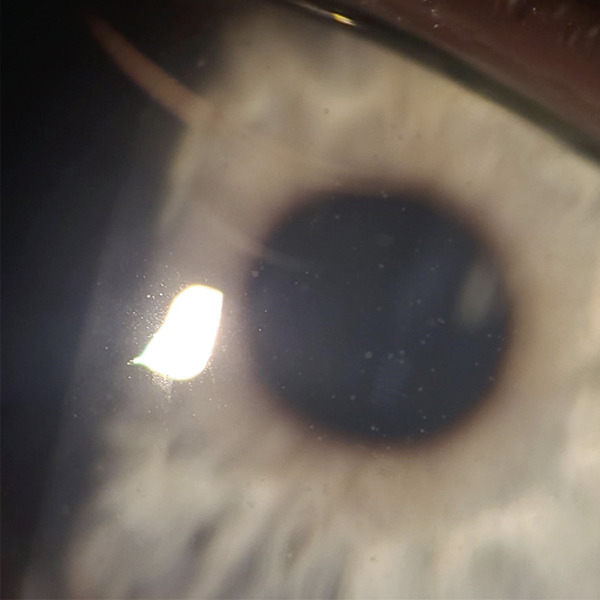
Representative image of primary subepithelial infiltrates (SEIs) from three of the four children.

**Figure 3:**
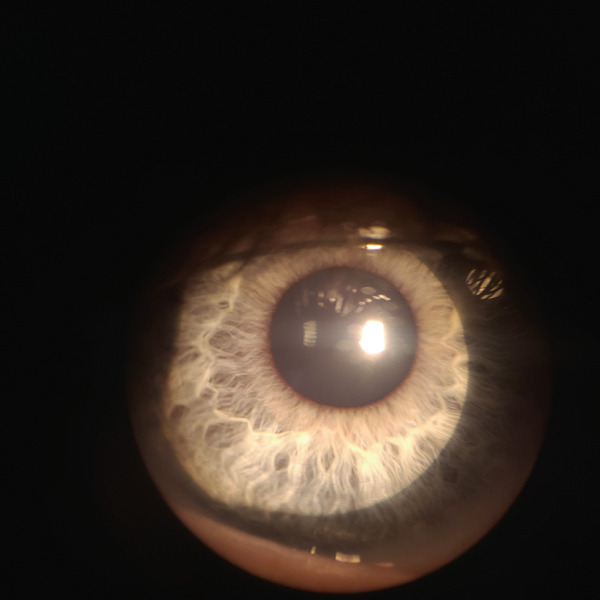
Slit lamp exam after subepithelial infiltrates (SEIs) have resolved.

Patient W is a 10‐year‐old male who presented in September complaining of a painful red eye. He reported a history of playing in a water‐filled ditch. Initial exam showed VA of 20/50 in the left eye with pinhole improvement to 20/40, IOP of 12 mmHg, 1+ conjunctival injection, 2+ conjunctival follicles, and scattered corneal subepithelial infiltrates (SEIs). PCR was positive for microsporidia and negative for other common pathogens such as adenovirus. The patient was then started on topical moxifloxacin 0.5% drops every 1 h. PCR was completed through HealthTrackRx (Denton, Texas). TaqMan chemistry assays and real‐time quantitative reverse transcription PCR (qRT‐PCR) were performed on the swabs. See attached for picture of results (Figure 1). The primers used were TaqMan chemistry primers developed by Thermo Fisher (Waltham, Massachusetts). The patient returned to the clinic 3 days later with improved VA to 20/25, improved conjunctival injection, and reduced numbers of SEIs. The moxifloxacin dose was adjusted to six times daily for 5 days, then four times daily. Four weeks after the initial presentation, VA improved to 20/20, the patient’s symptoms resolved, but less well‐defined SEIs appeared (Figure 2), so the patient was continued on moxifloxacin four times daily, and fluorometholone 0.1% drops were also prescribed four times daily. Three weeks later, VA was 20/15, and the SEIs were barely visible, so the patient was started on a steroid taper. Twelve weeks after initial presentation, the patient was no longer taking eye drops, was asymptomatic, and had a VA of 20/15 and a normal ocular exam with resolution of SEIs (Figure 3).

### 2.2. Case 2

Patient Z is a 23‐year‐old female who presented in December complaining of eye pain and redness in the left eye; she reported working as an aquatics instructor for children. On exam, VA was 20/60, IOP was 21 mmHg, and she had 3+ injection, 2+ chemosis, 2+ follicles, and 2+ poorly defined SEIs. She was treated for SEIs secondary to viral conjunctivitis with neomycin/polymyxin B/dexamethasone eye drops six times per day. Five days later, she presented with decreasing VA to 20/200 with no improvement on exam. PCR was performed and confirmed microsporidia, so polydex was tapered, and moxifloxacin 0.5% drops were initiated every hour. The patient was seen 2 weeks later due to travel. VA was 20/40, and there was 1+ injection, trace follicles, and scattered SEIs, so moxifloxacin was reduced to six times per day. One week later, the patient’s VA was 20/25 with persistent poorly defined SEIs, so moxifloxacin was reduced to four times daily, and fluorometholone was added four times daily. The patient was lost to follow‐up until her return 2 months later, at which point she stopped all her eye drops. VA was 20/30, IOP was 20 mmHg, and there were poorly defined SEIs. Fluorometholone 0.1% eye drops were restarted four times daily. The patient was lost to follow‐up again (Table 1).

## 3. Discussion

It is known that microsporidia are found throughout the environment and especially in the water [5]. Similar to previous reports of increased risk of microsporidiosis during the rainy season in Asia, this case series demonstrates that there may be increased risk during hurricane season in Southeastern Louisiana [8, 12, 13]. We were not aware of other reports in nearby facilities during the reported time period. The baseline incidence of microspoidiosis was difficult to determine as there was no formal surveillance data in Louisiana. Anecdotally, a nearby cornea specialist estimated three cases in the last 5 years in his practice. Other reports of contaminated water exposure include a case series from a practice in Georgia, a case series in Taiwan with hot springs exposure, a case series in India, and a case series in Singapore with soil exposure during a rugby tournament [3, 12, 13]. Thus, clinicians practicing in temperate climates should keep microsporidiosis high on their differential for viral keratoconjunctivitis, especially during periods of increased rainfall. Additionally, none of the patients presented here, nor any of the patients in the study by Huang et al. [3], were immunocompromised, underscoring the importance of vigilance in every patient. Children may be at increased risk, especially if they are actively playing in contaminated water sources.

PCR was used to confirm the diagnosis in all the presented cases which allowed for rapid diagnosis confirmation and rapid transition to appropriate therapy as shown in Case 5. In contrast, electron microscopy may take several days to receive the results. Further, in rural settings, such as in our cases, there may be limited access to timely direct microscopic examination. As the sensitivity and specificity of PCR testing for microsporidia continue to improve, greater emphasis should be placed on using PCR for diagnosis confirmation. This increases access to testing for patients and decreases the time to effective treatment from symptom presentation [6].

Treatment for our cases consisted of topical moxifloxacin 0.5% eye drops which resolved primary SEIs in all cases. As previously mentioned, typical treatment for microsporidia includes albendazole and fumagillin, with some new modalities including an azole plus a fluoroquinolone [11]. The advantage of moxifloxacin monotherapy is its ease of access. Other case series support the use of fluoroquinolones as the sole treatment or in combination with other traditional treatments for micropsoridial keratitis [14, 15]. Timely treatment is important for microsporidial stromal keratitis, but there have been studies suggesting that microsporidial keratoconjunctivitis may be self‐limited, particularly when steroid medication is stopped [4, 9]. A randomized controlled trial comparing polyhexamethylene biguanide 0.02% (PHMB) eye drops to placebo therapy in India demonstrated similar time to resolution in both groups [16]. While it may be reasonable to closely monitor patients rather than initiate PHMB therapy, it is unclear how placebo therapy compares to less toxic fluoroquinolone therapy. The findings are also limited by the performance of corneal debridement which may be therapeutic itself. Additionally, four patients had worsening symptoms in the placebo group which required treatment, so treatment is indicated in nonresolving cases. Further investigation is required to determine optimal microsporidial keratitis treatment. Based on our experience, we found initial frequent topical moxifloxacin instillation to be effective, every 1–2 h, while awake, depending on the severity of symptoms and extent of corneal lesions. There may be initial worsening of symptoms, but once the primary SEIs resolve and symptoms improve, the topical moxifloxacin can be tapered weekly over 4 weeks.

**Figure A1:**
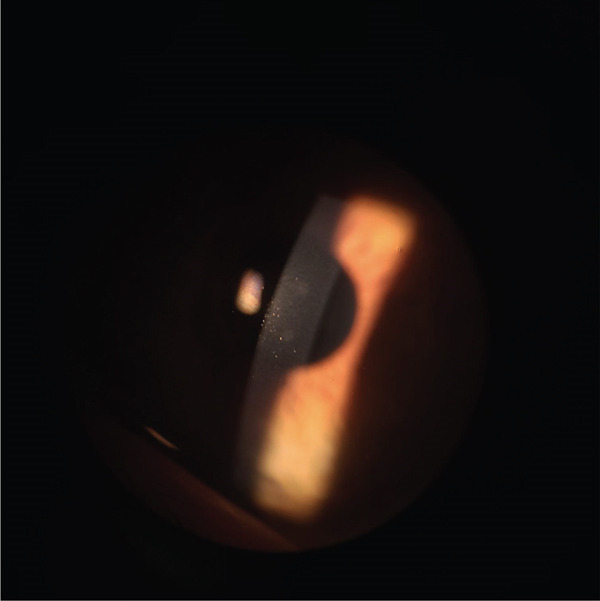
Secondary SEIs seen in one of the other patients.

**Figure A2:**
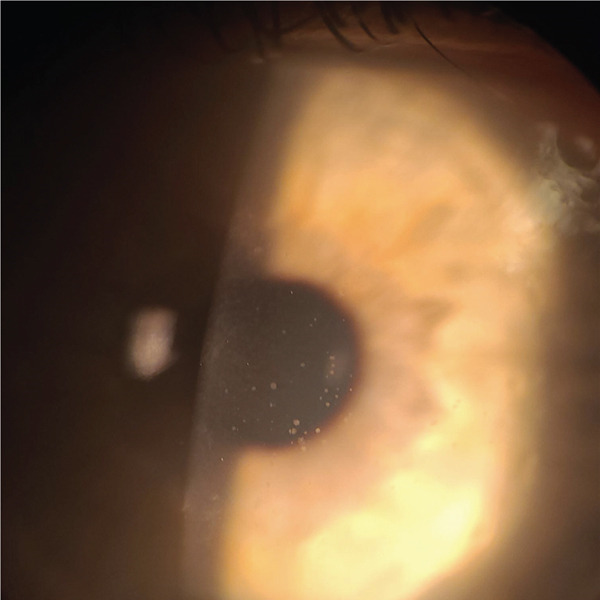
Primary SEIs seen in one of the other patients.

**Figure A3:**
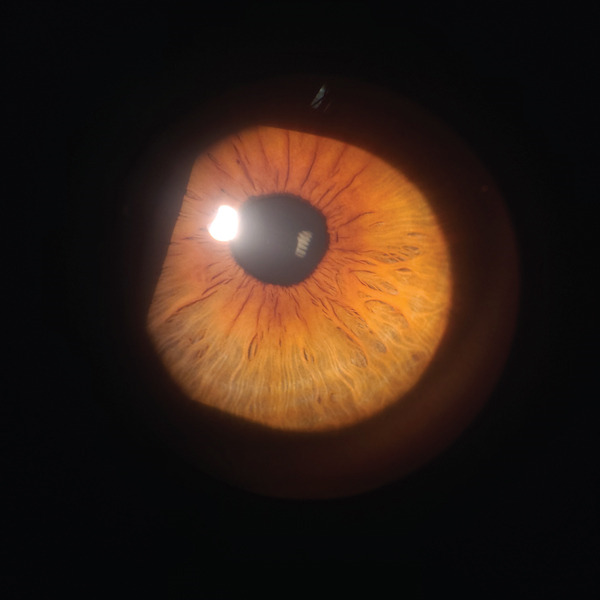
Additional example of resolution of SEIs from a patient seen in Figure A1.

Each patient in this series followed a similar clinical course. As patients were treated with moxifloxacin, the primary SEIs resolved completely, but secondary poorly defined SEIs appeared; more picture examples are seen in Appendix 1 (Figures A1, A2, and A3). The secondary SEIs responded well to topical steroid therapy and resolved completely in those who were adherent with follow‐up. A similar result was seen in the Ramatchandirane et al. [11] study where steroids were used due to corneal haziness. Treatment with steroids should be used only after the initial infection is controlled, as steroids may exacerbate the infection, as seen in our Case 2 and other reports in the literature [4]. We found it effective to target the ocular surface with fluoromethalone 0.1% four times daily once the secondary SEIs appeared. This can be tapered weekly over 4 weeks once SEIs resolve.

## 4. Conclusion

Ocular manifestations of microsporidia are increasingly being reported. Microsporidial keratoconjunctivitis is an important differential diagnosis of viral conjunctivitis in Southeastern Louisiana, and steroids should be used judiciously. Contaminated soil or water exposure is an important risk factor, especially in temperate climates during the rainy season such as the hurricane season of the Southern United States. PCR is useful for rapid diagnosis confirmation, allowing for faster initiation of effective treatment. Although treatment regimens vary widely, this case series demonstrates the efficacy of using moxifloxacin 0.5% initially followed by topical fluorometholone to treat secondary SEIs.

## Conflicts of Interest

The authors declare no conflicts of interest.

## Funding

No funding was received for this manuscript.

## Data Availability

The data that support the findings of this study are available from the corresponding author upon reasonable request.

## References

[1] Keeling P. J. and Fast N. M. , Microsporidia: Biology and Evolution of Highly Reduced Intracellular Parasites, Annual Review of Microbiology. (2002) 56, no. 1, 93–116, 10.1146/annurev.micro.56.012302.160854, 2-s2.0-0036406924, 12142484. 12142484

[2] Weber R. , Bryan R. T. , Schwartz D. A. , and Owen R. L. , Human Microsporidial Infections, Clinical Microbiology Reviews. (1994) 7, no. 4, 426–461, 10.1128/cmr.7.4.426, 2-s2.0-0028116395, 7834600. 7834600 PMC358336

[3] Huang A. S. , Cho J. S. , and Bertram B. A. , Microsporidial Keratitis Related to Water Exposure: A Case Series, Cureus. (2021) 13, no. 6, 10.7759/cureus.15760, e15760, 34164251. 34164251 PMC8214417

[4] Chan C. M. L. , Theng J. T. S. , Li L. , and Tan D. T. H. , Microsporidial Keratoconjunctivitis in Healthy Individuals: A Case Series, Ophthalmology. (2003) 110, no. 7, 1420–1425, 10.1016/s0161-6420(03)00448-2, 2-s2.0-0038154309, 12867402. 12867402

[5] Mota P. , Rauch C. A. , and Edberg S. C. , Microsporidia and *Cyclospora*: Epidemiology and Assessment of Risk From the Environment, Critical Reviews in Microbiology. (2000) 26, no. 2, 69–90, 10.1080/10408410091154192, 2-s2.0-0033923026, 10890351. 10890351

[6] Mena C. J. , Barnes A. , Castro G. , Guasconi L. , Burstein V. L. , Beccacece I. , Paulin P. C. , Arneodo J. , Carnevale S. , Astudillo G. , Cervi L. , Theumer M. G. , and Chiapello L. S. , Microscopic and PCR-Based Detection of Microsporidia Spores in Human Stool Samples, Revista Argentina de Microbiología. (2021) 53, no. 2, 124–128, 10.1016/j.ram.2020.04.005, 32595002. 32595002

[7] Mohanty A. , Sahu S. K. , Sharma S. , Mittal R. , Behera H. S. , das S. , and Lakhmipathy M. , Past, Present, and Prospects in Microsporidial Keratoconjunctivitis- A Review, Ocular Surface. (2023) 28, 364–377, 10.1016/j.jtos.2021.08.008, 34419638. 34419638

[8] Sharma S. , Das S. , Joseph J. , Vemuganti G. K. , and Murthy S. , Microsporidial Keratitis: Need for Increased Awareness, Survey of Ophthalmology. (2011) 56, no. 1, 1–22, 10.1016/j.survophthal.2010.03.006, 2-s2.0-78650194411, 21071051. 21071051

[9] Moshirfar M. , Somani S. N. , Shmunes K. M. , Espandar L. , Gokhale N. S. , Ronquillo Y. C. , and Hoopes P. C. , A Narrative Review of Microsporidial Infections of the Cornea, Ophthalmology and Therapy. (2020) 9, no. 2, 265–278, 10.1007/s40123-020-00243-z, 32157613. 32157613 PMC7196102

[10] Wei J. , Fei Z. , Pan G. , Weiss L. M. , and Zhou Z. , Current Therapy and Therapeutic Targets for Microsporidiosis, Frontiers in Microbiology. (2022) 13, 10.3389/fmicb.2022.835390, 35356517. PMC8959712 35356517

[11] Ramatchandirane B. , A M. , Marimuthu Y. , Nicodemus D. S. , and Yarra M. C. , Successful Treatment of Microsporidial Keratoconjunctivitis (MKC) With a Combination of Topical Voriconazole 1% and Gatifloxacin 0.5%: A Large Case Series of 29 Patients, Cureus. (2023) 15, no. 11, 10.7759/cureus.49247, e49247, 38143606. 38143606 PMC10743212

[12] Fan N.-W. , Wu C.-C. , Chen T.-L. , Yu W. K. , Chen C. P. , Lee S. M. , and Lin P. Y. , Microsporidial Keratitis in Patients With Hot Springs Exposure, Journal of Clinical Microbiology. (2012) 50, no. 2, 414–418, 10.1128/jcm.05007-11, 2-s2.0-84862966019, 22116156. 22116156 PMC3264156

[13] Tan J. , Lee P. , Lai Y. , Hishamuddin P. , Tay J. , Tan A. L. , Chan K. S. , Lin R. , Tan D. , Cutter J. , and Goh K. T. , Microsporidial Keratoconjunctivitis After Rugby tournament, Singapore, Emerging Infectious Diseases. (2013) 19, no. 9, 1484–1486, 10.3201/eid1909, 23965938. 23965938 PMC3810903

[14] Thanathanee O. , Athikulwongse R. , Anutarapongpan O. , Laummaunwai P. , Maleewong W. , Intapan P. M. , and Suwan-Apichon O. , Clinical Features, Risk Factors, and Treatments of Microsporidial Epithelial Keratitis, Seminars in Ophthalmology. (2016) 31, no. 3, 266–270, 10.3109/08820538.2014.962161, 2-s2.0-84939817326, 25495852. 25495852

[15] Loh R. S. , Chan C. M. L. , Ti S. E. , Lim L. , Chan K. S. , and Tan D. T. H. , Emerging Prevalence of Microsporidial Keratitis in Singapore: Epidemiology, Clinical Features, and Management, Ophthalmology. (2009) 116, no. 12, 2348–2353, 10.1016/j.ophtha.2009.05.004, 2-s2.0-70649096091, 19815287. 19815287

[16] Das S. , Sahu S. K. , Sharm S. , Nayak S. S. , and Kar S. , Clinical Trial of 0.02% Polyhexamethylene biguanide Versus Placebo in the Treatment of Microsporidial Keratoconjunctivitis, American Journal of Ophthalmology. (2010) 150, no. 1, 110–115, 10.1016/j.ajo.2010.01.038, 2-s2.0-77955675652, 20447613. 20447613

